# Evaluation of Risk Factors for Epilepsy in Pediatric Patients with Cerebral Palsy

**DOI:** 10.3390/brainsci10080481

**Published:** 2020-07-25

**Authors:** Małgorzata Sadowska, Beata Sarecka-Hujar, Ilona Kopyta

**Affiliations:** 1 Department of Pediatrics and Developmental Age Neurology, Upper Silesian Center for Child’s Health, 40-752 Katowice, Poland; m.sadowscy@gmail.com; 2 Department of Basic Biomedical Science, Faculty of Pharmaceutical Sciences in Sosnowiec, Medical University of Silesia in Katowice, 41-200 Sosnowiec, Poland; 3 Department of Pediatric Neurology, School of Medicine in Katowice, Medical University of Silesia in Katowice, 40-752 Katowice, Poland; ilonakopyta@autograf.pl

**Keywords:** cerebral palsy, children, seizures, drug-resistant epilepsy, risk factors

## Abstract

Cerebral palsy (CP) is a set of etiologically diverse symptoms that change with the child’s age. It is one of the most frequent causes of motor disability in children. CP occurs at a frequency of 1.5 to 3.0 per 1000 live-born children. CP often coexists with epilepsy, which is drug-resistant in a high number of cases. The aim of the present study was to analyze the associations between preconception, prenatal, perinatal, neonatal, and infancy risk factors for epilepsy in a group of pediatric patients with CP. We retrospectively analyzed 181 children with CP (aged 4–17 years at diagnosis), hospitalized at the Department of Pediatrics and Developmental Age Neurology in Katowice in the years 2008–2016. Division into particular types of CP was based on Ingram’s classification. Data were analyzed using STATISTICA 13.0 (STATSOFT; Statistica, Tulsa, OK, USA). Epilepsy was diagnosed in 102 children (56.35%), of whom 44 (43%) had drug-resistant epilepsy; only in 15 cases (14.71%) was epilepsy susceptible to treatment. The incidence of epilepsy varied between the types of CP. It occurred significantly more often in children with tetraplegia (75%), ataxic form (83%), and mixed form (80%) in comparison to diplegia (32%) and hemiplegia (38%). Maternal hypertension was found to be a risk factor for epilepsy in CP patients (OR = 12.46, *p* < 0.001) as well as for drug-resistant epilepsy (the odds ratio (OR) = 9.86, *p* = 0.040). Delivery by cesarean section increased the risk of epilepsy in the CP patients over two-fold (OR = 2.17, *p* = 0.012). We observed also that neonatal convulsions significantly increased the risk for epilepsy (OR = 3.04, *p* = 0.011) as well as drug-resistant epilepsy (OR = 4.02, *p* = 0.002). In conclusion, maternal hypertension, neonatal convulsions, and delivery by cesarean section were the most important factors increasing the risk of epilepsy as well as drug-resistant epilepsy in the analyzed group of patients with CP.

## 1. Introduction

Cerebral palsy (CP) is a group of permanent, but not unchanging, disorders of movement and/or posture and motor function, which are due to a nonprogressive interference, lesion, or abnormality of the developing/immature brain [1,2,3]. CP is one of the most frequent causes of motor disability in children [4,5,6,7,8,9]. Cerebral palsy occurs at a frequency of 1.5 to 3.0 per 1000 live-born children; these values change among selected groups of patients, depending on various risk factors [4]. Motor function disorders, which are the core symptoms of cerebral palsy, are frequently accompanied by other dysfunctions, such as sensation, perception, cognition, communication and behavioral disorders, secondary musculoskeletal disorders, and epilepsy [2,3,4].

Epilepsy is a separate, very important clinical problem in children with CP. The literature data on the morbidity of epilepsy in CP patients differ from one another. It is accepted that the general incidence of epilepsy in children and adults with CP varies widely between 15% and 55% in selected groups of patients and even reaches 90–94% depending on comorbidities and CP type [10,11,12,13,14,15,16]. The incidence of epilepsy in the group of 178 CP children evaluated by Zafeiriou et al. was 36.1% [17]. A similar prevalence was demonstrated in a group of 85 Chinese CP children (37.6%) [10]. On the other hand, a Brazilian retrospective analysis enrolling 100 CP patients hospitalized in tertiary referral hospitals indicated that epilepsy affected 62% of patients [18]. An analysis of records of 3424 children born between 1976 and 1998 from 17 regions of the Surveillance of Cerebral Palsy in Europe (SCPE) network showed a general incidence of epilepsy of 35% [19].

The incidence of epilepsy varies depending on the type of cerebral palsy. It is usually observed in tetraplegia, frequently accompanies hemiplegia, but seldom affects children with diplegia and the ataxic type of CP [14].

Numerous studies have analyzed the risk factors for epilepsy in children with CP. One of the significant factors is mental retardation [10,12,13,17,18]. Some of the studies also indicated a correlation between neonatal convulsions and a higher risk of epilepsy in children with CP [12,13,18].

The aim of the present study was to analyze possible correlations between preconception, prenatal, perinatal, and neonatal risk factors and epilepsy, with a special emphasis on drug-resistant epilepsy in a group of pediatric patients with cerebral palsy recruited from a single medical center in southern Poland.

## 2. Materials and Methods

### 2.1. Study Group

We retrospectively analyzed 181 children with CP (aged 4–17 years, 83 girls and 98 boys) hospitalized at the Department of Pediatrics and Developmental Age Neurology in Katowice in 2008–2016. The study had a retrospective character and consisted of an analysis of the patients’ records. A diagnosis of cerebral palsy was based on the definition published earlier [1,2,3].

The inclusion criteria for the analyzed group were as follows:

- Aged between 4 and 17 years at the time of hospitalization/enrolment,
- The diagnosis of cerebral palsy verified by an experienced pediatric neurologist,
- Available neuroimaging: magnetic resonance imaging (MRI) or computed tomography (CT) results.

The exclusion criteria were as follows:

- Age younger than 4 or over 17,
- Lack of neuroimaging results,
- Clinical features of progressive encephalopathies,
- Metabolic inborn errors.

The Local Ethical Committee gave an opinion on the study (decision no. KNW/0022/KB/179/16).

### 2.2. Data Extraction from Medical Records

The following data were extracted according to the ICD-10 classification; we focused on G80 patients’ records from the hospital data system: data on family history, pregnancy and delivery, types of CP, severity of CP, the level of psychological development, neuroimaging results, data regarding epilepsy (treatment; factors related to the preconception, prenatal, perinatal, and neonatal periods that could be associated with epilepsy; seizure outcome).

The assumed risk factors of epilepsy in children with CP were divided into the following:

- Preconception and prenatal (mother’s age, mother’s diseases, family history of epilepsy, mother’s reproductive history, pregnancy order, multiple pregnancy incidence, bleeding from the genital tract during gestation, arterial hypertension during gestation, infections during pregnancy, preterm contractions, abruptio placentae, pregnancy duration);
- Perinatal and postnatal (type of delivery, birth weight, Apgar score at first and fifth minute, neonatal seizures, respiratory insufficiency, infections in the neonatal period, intracranial bleeding).

### 2.3. Key Classifications and Definitions

Division into CP types (diplegia, hemiplegia, tetraplegia, extrapyramidal form, ataxic form, and mixed form) was based on Ingram’s classification [20]. The level of movement disorder severity according to the Gross Motor Function Classification Scale (GMFCS) was subjected to analysis [21].

The MRI brain images were classified according to the Magnetic Resonance Imaging Classification System (MRICS) recommended by SCPE [22]. According to MRICS, brain images in children with CP are classified into five groups: A. Maldevelopments: A.1. Disorders of cortical formation, A.2. Other maldevelopments; B. Predominant white matter injury: B.1. Periventricular leukomalacia (PVL), B.2. Sequelae of intraventricular hemorrhage (IVH) or periventricular hemorrhagic infarction, B.3. Combination of PVL and IVH sequelae; C. Predominant grey matter injury: C.1. Basal ganglia/thalamus lesions, C.2. Cortico-subcortical lesions not covered under C3, C.3. Arterial infarctions; D. Miscellaneous; E. Normal [22].

According to the information contained in the medical records of patients included in the study, the intellectual developmental level was evaluated by psychologists in 168 out of 181 patients by the following psychological methods: Wechsler Intelligence Scale for Children—Revised (WISC-R), Wechsler Adult Intelligence Scale—Revised (WAIS-R), Psyche Cattell Infant Intelligence Scale, Columbia Mental Maturity Scale (CMMS), Leiter International Performance Scale, Intelligence and Development Scales—Preschool (IDS-P) as well as the H.C. Gunzburg Scale of Measurement of Social Development in children with mental disability.

Based on the results of psychological tests, the recruited children were divided into the following five groups:

- Children with standard psychological assessment or below standard;
- Children with mild impairment;
- Children with moderate impairment;
- Children with significant impairment;
- Children with deep impairment.

The diagnosis of epilepsy was followed by the definition formulated by the International League Against Epilepsy (ILAE) task force [23,24]. In our study, the group with drug-resistant epilepsy was separate. The definition of drug-resistant epilepsy was also adopted, after the ILAE, as “failure of adequate trials of two tolerated, appropriately chosen and used antiepileptic drug schedules (whether as monotherapies or in combination) to achieve sustained seizure freedom.” Seizure freedom is considered to be when the patient is seizure-free for more than one year or has sporadic seizures separated by a period three times longer than the longest interval between seizures prior to the treatment, whichever is longer. Drug-responsive epilepsy is if a patient with epilepsy has been seizure-free for a minimum of 3 times the longest pretreatment interseizure interval or 12 months, whichever is longer. If the course of person’s epilepsy does not fulfill the definition criteria for either drug-resistant or drug-responsive epilepsy, the drug responsiveness is classified as “undefined” [25,26,27].

### 2.4. Statistical Analysis

STATISTICA 13.0 software (STATSOFT; Statistica, Tulsa, OK, USA) was used to perform the statistical analysis. For continuous variables, mean values (M) and standard deviations (SD) were estimated. For categorical variables, absolute numbers (n) and relative numbers (%) were assessed. To compare continuous variables, the Mann‒Whitney U test was used. A stochastic independence χ2 test with Yates’s correction was used to compare categorical variables. The strength of the relationship of selected parameters (maternal age, maternal systemic diseases, burdened obstetric history, family history of epilepsy, pregnancy order, sustained pregnancy, premature contractions, premature placental abruption, premature departure of the fetal waters, other pregnancy pathologies, type of delivery, birth weight, scoring on the Apgar scale at 1 and 5 min, neonatal convulsions, respiratory failure, intraventricular hemorrhage, neonatal and infantile infections) with epilepsy or drug-resistant epilepsy were determined by calculating the odds ratio (OR) with a 95% confidence interval (CI) using univariate and a multivariate logistic regression model, with adjusted factors. A value of *p* ≤ 0.05 was considered to be statistically significant.

## 3. Results

### 3.1. Characteristics of the Study Group

The general characteristics of the CP patients in terms of sex, age, birth weight, type of cerebral palsy, GMFCS classification, psychological assessment result, and type of changes in the MRI are presented in Table 1.

**Figure 1:**
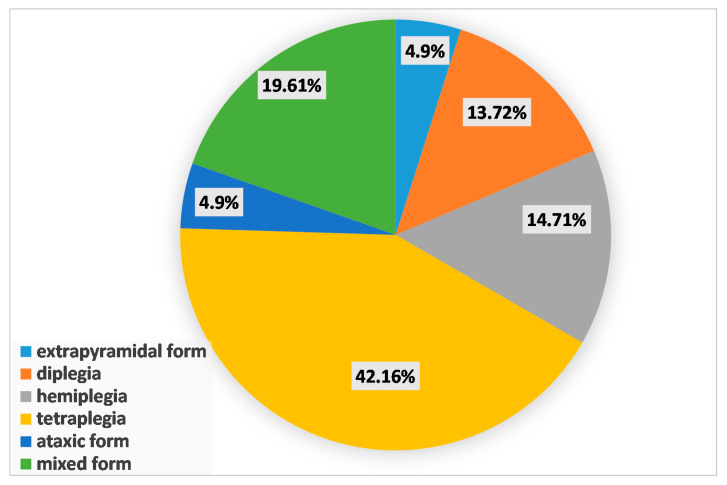
Distribution of types of cerebral palsy according to Ingram’s classification [20] in patients with epilepsy.

Among the recruited patients, epilepsy was diagnosed in 102 children (i.e., 56.35%). In the total group of analyzed CP patients, males were slightly more prevalent. A similar male/female ratio was observed in the group of CP patients without epilepsy. The age of the examined children, as well as their birth weight, did not differ significantly between the examined subgroups. However, the form of CP differed significantly between the analyzed patient subgroups. In the epilepsy subgroup, the most common form of CP was tetraplegia (42%), and in the nonepileptic subgroup, diplegia and hemiplegia were most common (in 38% and 30% of patients, respectively). Mixed CP was more common in the epilepsy subgroup (19.61%) compared to the nonepileptic subgroup (6.33%). Figure 1 presents the group of CP and epilepsy patients with regard to the cerebral palsy type according to Ingram’s classification.

**Figure 2:**
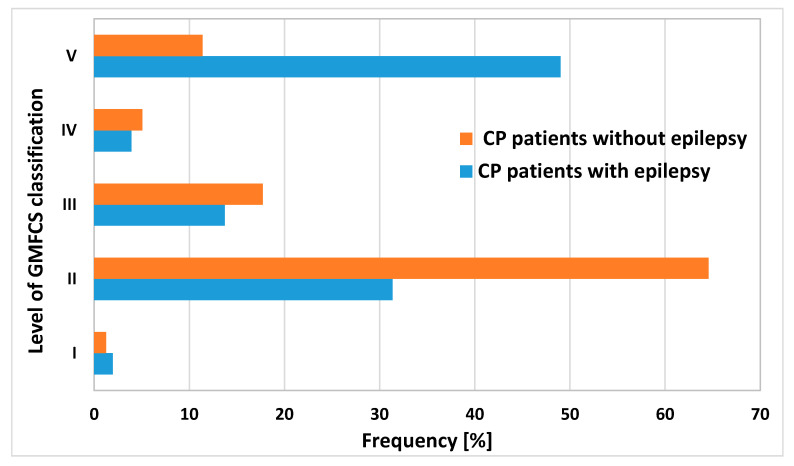
The distribution of GMCSF levels in children with and without epilepsy. GMFCS—Gross Motor Function Classification Scale; CP—cerebral palsy.

Significant differences were observed in the GMFCS classification. In the group of children with epilepsy, the degree of motor disability of patients evaluated on the GMFCS scale was most often assessed as level V or II (49% and 31%, respectively), and in the nonepileptic subgroup most children were assigned to level II (65%) (Figure 2).

**Figure 3:**
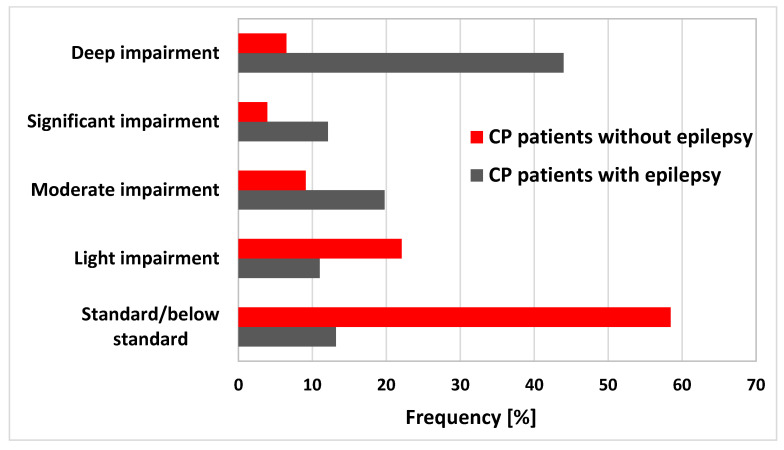
The distribution of intellectual disabilities in CP patients with and without epilepsy. CP—cerebral palsy.

The results of the psychological assessment differentiated the two subgroups examined: with and without epilepsy. Children with epilepsy were more deeply disabled in their intellectual level (44% vs. 6% in the group without epilepsy). In turn, for children without epilepsy, intellectual development was at the age norm or at a level below the average (58% of children vs. 13% in the group with epilepsy) (Figure 3).

In 177 children (i.e., 97.79%), MRI results were analyzed according to the MRICS classification. There were no significant differences in the incidence of individual types of lesions in the MR study between the analyzed subgroups of children with CP.

### 3.2. Analysis of Preconception and Prenatal Risk Factors for Epilepsy in CP

The incidence of preconception and prenatal risk factors for epilepsy, with a particular emphasis on drug-resistant epilepsy, is shown in Table 2. It was observed that only maternal hypertension during pregnancy differs significantly in patients with CP and epilepsy from patients with nonepileptic CP (15% vs. 1%).

Univariate logistic regression revealed that hypertension may be a risk factor for epilepsy in patients with CP (OR = 12.46, 95% CI 1.57–98.59, *p* < 0.001). Other factors occurred with similar frequency in both subgroups. In the case of drug-resistant epilepsy, it was found that the factors differentiating the group with drug-resistant epilepsy from the group of children without epilepsy are: systemic diseases of the mother (i.e., asthma, congenital heart defect, cardiac rhythm disturbances, thyroid diseases, cancer, rheumatologic diseases, mental health problems, intellectual disability), the order of pregnancy, hypertension during gestation, and premature placental abruption. Systemic diseases of the mother were more common in children without epilepsy (19% vs. 5% in children with drug-resistant epilepsy). In the case of pregnancy order, children with drug-resistant epilepsy were more likely to have been the first pregnancy (62%) than children without epilepsy (42%).

Hypertension during pregnancy was more common in the group with drug-resistant epilepsy than in the group without epilepsy (12% vs. 1%). On the other hand, premature placental abruption is more common in children without epilepsy (14%) than in patients with drug-resistant epilepsy (2%). Hypertension during pregnancy significantly increased the risk of drug-resistant epilepsy in children with CP (OR = 9.86, 95% CI 1.08–89.63, *p* = 0.040). It was also observed that there was a family history of epilepsy in a greater number of children with drug-resistant epilepsy than in children without epilepsy (11% vs. 7%); however, the difference was only borderline significant (*p* = 0.052).

From the whole studied group of 181 patients with CP, family history of epilepsy affected 10 children, i.e., 6%. Epilepsy most often occurred in siblings or other family members. Only one mother suffered from epilepsy (with traumatic history): she was treated during pregnancy with Levetiracetam and seizures occurred sporadically. The child was born by cesarean section at 36 weeks of pregnancy, with normal body weight and a full score on the Apgar scale, and presented the diplegic form of CP; in his sixth year he suffered from epilepsy with generalized onset seizures. After first-line monotherapy, the seizures resolved; epilepsy was found with transient drug susceptibility (observation period: three months).

### 3.3. Analysis of the Perinatal, Neonatal, and Infant-Related Risk Factors for Epilepsy in CP

The type of delivery and neonatal convulsions were significantly different in the subgroup of CP children with epilepsy compared to CP children without epilepsy. In the group with epilepsy, birth was more often by cesarean section (56%), while in the group with drug-resistant epilepsy it was 52%. In the nonepileptic group, vaginal birth (63%) was most commonly observed (Table 3).

Univariate analysis showed that delivery by cesarean section increased the risk of epilepsy in the studied group of patients with CP over 2-fold (OR = 2.17, 95% CI 1.17–4.02, *p* = 0.012). In the whole group of patients with CP and epilepsy, neonatal seizures occurred in 37%, while in the group without epilepsy the frequency was 14% (*p* = 0.001). Convulsions in the neonatal period were most commonly observed in the group of children with CP and drug-resistant epilepsy (40%). Univariate analysis showed that neonatal convulsions significantly increased the risk of epilepsy (OR = 3.04, 95% CI 1.28–7.21, *p* = 0.011) as well as drug-resistant epilepsy (OR = 4.02, 95% CI 1.64–9.85, *p* = 0.002).

## 4. Discussion

In the present study, CP was most often present in boys (male/female ratio = 1.18:1, i.e., 98 boys vs. 83 girls). In population-based trials, the higher incidence of CP in boys was confirmed [28,29]. The proportions of the given CP types in our study differ from other data. Results similar to ours were presented in the study by Kułak et al. [30].

In our study group, epilepsy was diagnosed in 102 children out of 181 (i.e., 56.35%). Such a high percentage of patients with cerebral palsy and concomitant epilepsy may result from the fact that we recruited hospitalized patients, and often the reason for hospitalization was continued seizures and the need for correction of antiepileptic therapy. However, a similar percentage of epilepsy was described by Turkish authors in a group of 98 children with CP [13]. In the analyzed group of children with CP and epilepsy, the largest number of children presented tetraplegia (42%). Children with CP and epilepsy exhibited the highest degree of motor disability, as evaluated by the GMFCS (in 49% of them), as well as the most severe intellectual disability (in 44%). In turn, in the subgroup of CP children without epilepsy, the dominant types were diplegia and hemiplegia (38% and 30%, respectively), grade II motor dysfunction as evaluated by the GMFCS (65%), and intellectual development in the normal range or slightly below normal (in 58% of patients). These results are comparable to the data recorded by other researchers [31,32].

A population-based study conducted in Australia on a group of 3466 children with CP born between 1996 and 2005 indicated that the incidence of epilepsy increased with the degree of motor disability; it was highest in tetraplegic patients (53%) and lowest in diplegic patients (14%) [33]. In the Turkish study, the intellectual development of CP children depending on coexisting morbidities was analyzed. The authors confirmed a statistically significant association between epilepsy and intellectual developmental level. In 68% of patients with epilepsy, the intellectual developmental delay was severe [34]. In a group of preschool CP children from Iceland, epilepsy was found to be the only factor of all the analyzed comorbidities influencing the level of intellectual development. Moreover, in the epilepsy subgroup the degree of motor disability evaluated by GMFCS was IV or V [35]. An Australian population-based study of over 1000 CP children indicated that the coexistence of epilepsy is associated with a high incidence of intellectual disability (79%) [36]. Carlson et al. conducted research on 46 CP children aged six to 14 years and born in 1987–1994 in the Goteborg area. The epilepsy incidence in this group was 38%. All tetraplegic patients and about one-third of patients with the dyskinetic, diplegic, and hemiplegic types of CP presented with epilepsy, while only two out of 11 children with the atactic form were diagnosed with coexisting epilepsy. The authors concluded that the incidence of epilepsy had a negative correlation with the level of intellectual development: just 19% of children with a normal developmental level suffered from epilepsy, while 61% of children had a severe mental disability [37].

Among the children with CP, 44 had drug-resistant epilepsy (out of an overall 102 with epilepsy (i.e., 43%)). The highest incidence of intractable epilepsy was found in patients with mixed form (55%), tetraplegic form (51%), and extrapyramidal form (80%), while hemiplegia was found in only five children with drug-resistant epilepsy.

In our patients, antiepileptic drug (AED) withdrawal after a three-year seizure-free period was successful in nine children (8.8%); however, in four children, seizure recurrence was observed. Zefeiriou et al. reported that, out of 178 CP + epilepsy children, up to 75.3% were seizure-free after the three-year period and treatment could be withdrawn; in 18 children, seizures recurred [17]. The study by Delgado et al. [38] demonstrated a two-year seizure-free period in 12.9% of patients; the seizures recurred in 41.5% of children after AED withdrawal. The highest recurrence ratio was found in children with hemiplegia (61.5%) compared to diplegia (14.3%).

In our study group, epilepsy amenable to treatment was diagnosed in 15% of children, epilepsy with undetermined vulnerability was diagnosed in 42%, and drug-resistant epilepsy was diagnosed in 43%. Kulak and Sobaniec noted a good response to antiepileptic treatment in nearly half of the analyzed CP children, i.e., being seizure-free for at least a two-year period [12]. Brazilian authors studied a group of 100 CP children with coexisting epilepsy in 62 of them; 53% (33 children) were seizure-free for at least one year [18]. In the research by Gururaj et al., a good response to epilepsy treatment was described in 21% of children [11].

We used the up-to-date definition of drug-resistant epilepsy, which is different to that used by the authors cited above. We also used the terms “epilepsy amenable to treatment” and “epilepsy with undetermined vulnerability to treatment,” according to the new ILAE guidelines [25]. The duration of studies and the differences in the definitions used makes it hard to compare results. However, the number of children suffering from drug-resistant epilepsy in our study was similar to the results of Kułak and Sobaniec [12], whereas the number of children with epilepsy amenable to treatment was similar to that reported by Gururaj et al. [11].

Our results, similar to data collected by Kułak and Sobaniec, did not prove an association between pregnancy duration and epilepsy occurrence [12]. In studies by Zelnik et al. [37] and Carlson et al. [39], the number of CP children with epilepsy was higher in the group born on time.

In an American study, 889 out of 966 children born before 28 weeks of gestation were evaluated at age two and again at age 10 [40]. By age 10, at least one seizure had been observed in 12.2% of the group, and epilepsy was diagnosed in 7.6%. The authors conclude that the risk of epilepsy in this group was 7–14 times higher than in the general population and the main risk factor was the duration of the pregnancy—the shorter the pregnancy duration, the higher the risk of seizures and epilepsy. In three-quarters of the group, the seizure onset was in the first year of life and two-thirds of children had comorbidities like intellectual developmental delay, autistic spectrum disorders, attention deficit disorder, and, on neuroimaging, abnormalities of white matter [40]. Of our patients, 14 (i.e., 7.86%) were born before 28 weeks of gestation; in nine of them, epilepsy was diagnosed and two met the criteria for drug-resistant epilepsy.

We also analyzed the associations with the newborn condition, as evaluated at the first and fifth minutes of life; we did not check it at the 10th minute due to a lack of data in our retrospective material. We did not find an association between the evaluations done at the first and fifth minutes and epilepsy occurrence. The results of Swedish research on children born on time, however, proved that the risk of CP increases 425.5-fold in children with three points on the Apgar score at the 10th minute compared to children with 10 points. Therefore, the risk of epilepsy occurrence appears to depend on the Apgar evaluation at the 10th minute [41]. Zelnik et al. [39] proved associations between a low Apgar score (0–3 points) at the first and fifth minutes and epilepsy, as well as between neonatal seizures and epilepsy. The latter conclusion corroborates our results and those of Gururaj et al. [11]. The latter authors also conclude that satisfactory seizure control in CP children is more likely in patients without neonatal seizures in their history and with seizure onset after the first year of life [11]. In Swedish data, the occurrence of neonatal seizures was associated with epilepsy onset in CP patients, while Chinese authors did not confirm such a dependence [10,37]. In research by Kułak and Sobaniec (cited previously), the factors associated with epilepsy onset in 198 CP children were as follows: low birth weight (<2500 g), neonatal seizures, seizure onset before the first year of life, positive family history of epilepsy, and degree of motor as well as intellectual disability [12]. These results are somewhat consistent with ours; we did not confirm the associations of epilepsy with the newborn’s gender, body mass at birth, and pregnancy order, but the latter factor was meaningful in the context of drug-resistant epilepsy. Among the prenatal factors, only arterial hypertension at gestation was found to be a meaningful risk factor for epilepsy. The population-based study conducted in Denmark on a cohort of 1.5 million children born from single gestations proved an association between eclampsia and epilepsy, especially in children born on time or later—but not in children born prematurely [42]. In a Swedish population-based study by Razaz et al. carried out on children born between 1997 and 2011, epilepsy was diagnosed in 0.5%. The authors observed that meaningful risk factors for epilepsy onset were the mother’s BMI, pre-eclampsia, Apgar score below seven points at the fifth minute, and neonatal seizures [43].

A meta-analysis performed on a sizeable group of CP patients and controls found that any type of cesarean delivery (elective or emergency) was associated with cerebral palsy in term but not in preterm newborns (OR = 1.6 and OR = 0.81, respectively) [44]. Previously, a study conducted in three Danish counties demonstrated that, in children born by cesarean section (adjusted for birth weight, presentation, malformations, and county), the rate of epilepsy was 1.4, similar to the rate for birth at 42 weeks (1.3) [45]. In our study, cesarean section was among the postnatal risk factors for epilepsy. It is worth underlining that the cesarean section itself is not a risk factor for epilepsy in CP children, but rather the indications to this procedure, i.e., bleeding, eclampsia, or premature rupture of membranes, that have an influence on the newborn condition and also, potentially, subsequent psychomotor development and comorbidities. On the other hand, Zelnik et al. [39] found that vaginal delivery was more common in children with epilepsy, whereas cesarean section was more common in children without epilepsy. In turn, Kułak and Sobaniec [12] did not find an association between epilepsy in CP children and delivery by cesarean section. As recently observed, cesarean delivery may have an influence on infant brain development, including lower white matter development in widespread brain regions and lower functional connectivity in the brain [46].

The weakness of this study is the low number of the enrolled patients as well as the fact that they were patients hospitalized in a tertiary referral pediatric neurology department. Thus, the recruited patients do not represent a typical population of CP children as at least some of them were admitted to hospital for antiepileptic therapy modification after unsatisfactory results. This is why the number of epileptic children as well as the incidence of drug-resistant epilepsy is so high. Children with extrapyramidal, as well as cerebellar types, were underrepresented, which influenced the results. Another research limitation is its retrospective character, which is the reason for the limited data and factors we could evaluate as we focused on these available in the patients’ records.

## 5. Conclusions

The risk factors associated with epilepsy onset in the analyzed group of children were arterial hypertension during pregnancy, delivery by cesarean section and neonatal seizures, and, for drug-resistant epilepsy, maternal arterial hypertension, neonatal seizures, and positive family history of epilepsy. The knowledge of risk factors of epilepsy in children at risk of CP requires special care and behavioral observation, as well as recurrent evaluation by EEG. If epilepsy occurs in CP children, the knowledge of drug resistance provides a prognosis of the disease and should be a reason for therapy intensification and the consideration of nonpharmacological treatments. All children with a diagnosis of CP, but especially these at higher risk, should be thoroughly observed for potential epilepsy occurrence to initiate early treatment, which may be crucial to enable the better development of these patients.

## Figures and Tables

**Table 1:** Characteristics of the study group.

	Total Group with CP	Subgroup with CP and Epilepsy	Epilepsy-Free Subgroup	*p*
	*n* = 181	*n* = 102	*n* = 79	
**Sex, *n* (%)**				0.565
Girls	83 (45.86)	49 (48.04)	34 (43.04)	
Boys	98 (54.14)	53 (51.96)	45 (56.96)	
**Age at Enrollment to Study Group (Years),** M ± SD	8.72 ± 4.02	9.05 ± 4.02	8.29 ± 4.01	0.215
Median (Min.–Max.)	7.0 (4.0–17.0)	8.5 (4.0–17.0)	7.0 (4.0–17.0)	
**Birth weight (g)** M ± SD	2427.5 ± 982.9	2452.6 ± 1018.3	2394.9 ± 940.6	0.82
Median (Min.–Max.)	2500 (600–4810)	2600 (600–4810)	2400 (600–4300)	
**Type of CP acc. to Ingram’s Classification, n (%)**				**<0.001**
Diplegia	44 (24.31)	14 (13.72)	30 (37.97)	
Hemiplegia	39 (21.55)	15 (14.71)	24 (30.38)	
Tetraplegia	57 (31.49)	43 (42.16)	14 (17.72)	
Extrapyramidal Form	10 (5.52)	5 (4.90)	5 (6.33)	
Cerebellar Form	6 (3.31)	5 (4.90)	1 (1.27)	
Mixed Form	25 (13.81)	20 (19.61)	5 (6.33)	
**GMFCS Classification, n (%)**				**<0.001**
I	3 (1.65)	2 (1.96)	1 (1.26)	
II	83 (45.86)	32 (31.37)	51 (64.56)	
III	28 (15.47)	14 (13.73)	14 (17.72)	
IV	8 (4.42)	4 (3.92)	4 (5.06)	
V	59 (32.60)	50 (49.02)	9 (11.39)	
**Psychological Assessment** (missing data *n* = 13), n (%)				**<0.001**
Standard/Below Standard	57 (33.93)	12 (13.19)	45 (58.44)	
Light Impairment	27 (16.07)	10 (10.99)	17 (22.08)	
Moderate Impairment	25 (14.88)	18 (19.78)	7 (9.09)	
Significant Impairment	14 (8.33)	11 (12.09)	3 (3.90)	
Deep Impairment	45 (26.79)	40 (43.96)	5 (6.49)	
**Type of changes in MRI acc. to MRICS, n(%)** (missing data *n* = 4)				0.482
A.1.	8 (4.52)	2 (1.98)	6 (7.98)	
A.2.	12 (6.78)	8 (7.92)	4 (5.26)	
B.1.	56 (31.65)	30 (29.70)	26 (34.21)	
B.2.	10 (5.65)	6 (5.94)	4 (5.26)	
B.3.	37 (20.90)	22 (21.78)	15 (19.74)	
C.1.	1 (0.56)	1 (0.99)	0 (0.00)	
C.2.	5 (2.82)	5 (4.95)	0 (0.00)	
C.3.	10 (5.65)	5 (4.95)	5 (6.58)	
D	17 (9.60)	9 (8.91)	8 (10.53)	
E	11 (6.21)	5 (4.95)	6 (7.89)	
Mixed	10 (5.65)	8 (7.92)	2 (2.63)	

CP—cerebral palsy; M—mean value; SD—standard deviation; GMFCS—Gross Motor Function Classification Scale; MRI—Magnetic Resonance Imaging; MRICS—Magnetic Resonance Imaging Classification System; The bold shows statistical significance.

**Table 2:** Preconception and prenatal risk factors, depending on the occurrence of epilepsy.

	Total Group	Epilepsy-Free Subgroup	Subgroup with CP and Epilepsy	*p*	Subgroup with CP and Drug-Resistant Epilepsy	*p* *
**Mother’s age, *n* (%)**				0.58		0.155
<20 Years	13 (7.78)	7 (9.59)	6 (6.38)		4 (10.00)	
20–34 Years	123 (73.65)	53 (72.60)	70 (74.47)		34 (85.00)	
≥35 Years	31 (18.56)	13 (17.81)	18 (19.15)		2 (5.00)	
Mother’s Systemic diseases, *n* (%) **				0.118		**0.040**
Yes	24 (13.95)	14 (18.67)	10 (10.31)		2 (4.88)	
No	148 (86.05)	61 (81.33)	87 (89.69)		39 (95.12)	
**Burdened Obstetric History, *n* (%)**				0.181		0.074
Yes	42 (24.00)	22 (28.95)	20 (20.20)		6 (14.29)	
No	133 (76.00)	54 (71.05)	79 (79.80)		36 (85.71)	
**Family History of Epilepsy, *n* (%)**				0.138		0.052
Yes	10 (5.68)	2 (6.67)	8 (7.92)		5 (11.36)	
No	166 (94.32)	73 (97.33)	93 (92.07)		39 (88.64)	
**The Order of Pregnancy, *n* (%)**				0.699		**0.017**
I	78 (44.32)	32 (41.56)	46 (46.46)		26 (61.90)	
II	45 (25.57)	18 (23.38)	27 (27.27)		11 (26.19)	
III	30 (17.04)	19 (24.66)	11 (11.11)		3 (7.14)	
IV	12 (6.82)	4 (5.19)	8 (8.08)		1 (2.38)	
V or More	11 (6.25)	4 (5.19)	7 (7.07)		1 (2.38)	
**Multiple Pregnancy, *n* (%)**				0.615		0.704
Yes	14 (7.82)	7 (8.97)	7 (6.93)		3 (6.98)	
No	165 (92.18)	71 (91.03)	94 (93.07)		40 (93.02)	
**Duration of Pregnancy, *n* (%)**				0.431		0.21
< 28 Hbd	14 (7.86)	5 (6.33)	9 (9.09)		2 (4.88)	
28–32	35 (19.66)	20 (25.32)	15 (15.15)		5 (12.19)	
32–37	46 (25.84)	20 (25.32)	26 (26.26)		13 (31.71)	
37–42	80 (44.94)	32 (40.51)	48 (48.48)		20 (48.78)	
≥42 Hbd	3 (1.68)	2 (2.53)	1 (1.01)		1 (2.44)	
**Hypertension in Pregnancy, *n* (%)**				**<0.001**		**0.04**
Yes	15 (8.82)	1 (1.35)	14 (14.58)		5 (11.90)	
No	155 (91.17)	73 (98.65)	82 (85.42)		37 (88.09)	
**Genital Tract Bleeding during Pregnancy, *n* (%)**				0.115		0.181
Yes	24 (14.12)	14 (18.92)	10 (10.42)		4 (9.52)	
No	146 (85.88)	60 (81.08)	86 (89.58)		38 (90.48)	
**Infections during Pregnancy, *n* (%)**				0.524		0.743
Yes	33 (19.41)	16 (21.62)	17 (17.71)		8 (19.05)	
No	137 (80.59)	58 (78.38)	79 (82.29)		34 (80.95)	
**Pregnancy Supported, *n* (%)**				0.968		0.576
Yes	37 (21.76)	16 (21.62)	21 (21.87)		11 (26.19)	
No	133 (78.23)	58 (78.38)	75 (78.13)		31 (26.19)	
**Premature Contractions, *n* (%)**				0.405		0.933
Yes	30 (17.65)	11 (14.86)	19 (19.79)		6 (14.29)	
No	140 (82.35)	63 (85.14)	77 (80.21)		36 (85.71)	
**Premature Placental Abruption, *n* (%)**				0.278		**0.05**
Yes	18 (10.59)	10 (13.51)	8 (8.33)		1 (2.38)	
No	152 (89.41)	64 (86.49)	88 (91.67)		41 (97.62)	
**Premature Departure of Amniotic Fluid, *n* (%)**				0.451		0.986
Yes	28 (16.47)	14 (18.92)	14 (14.58)		8 (19.05)	
No	142 (83.53)	60 (81.08)	82 (85.42)		34 (80.95)	
**Others, *n* (%)**				0.21		0.168
Yes	45 (26.47)	16 (21.62)	29 (30.21)		14 (33.33)	
No	125 (73.53)	58 (78.38)	67 (69.79)		28 (66.67)	

CP—cerebral palsy; * In comparison to CP patients without epilepsy; ** Mother’s systemic diseases: asthma, congenital heart defect, cardiac rhythm disturbances, thyroid diseases, cancer, rheumatologic diseases, mental health problems, intellectual disability. Data on pregnancy are missing in 11 cases.

**Table 3:** Perinatal, neonatal, and infant-related risk factors for epilepsy in CP.

	Total Group	Epilepsy-Free Subgroup	Subgroup with CP and Epilepsy	*p*	Subgroup with CP and Drug-Resistant Epilepsy	*p* *
**Type of Delivery, *n* (%)**				**0.012**		0.098
Vaginal Birth	93 (52.84)	50 (63.29)	43 (44.33)		20 (47.62)	
Cesarean Section	83 (47.16)	29 (36.71)	54 (55.67)		22 (52.38)	
**Birth Weight, *n* (%)**				0.654		0.281
<1000 g, ELBW	16 (9.04)	4 (5.19)	12 (12.00)		4 (9.30)	
1000–1499 g, VLBW	25 (14.12)	14 (18.18)	11 (11.00)		5 (11.63)	
1500–2499 g, LBW	45 (25.43)	22 (28.57)	23 (23.00)		7 (16.28)	
2500–4000 g,	85 (48.02)	35 (45.45)	50(50.00)		26 (60.46)	
>4000 g	6 (3.39)	2 (2.60)	4 (4.00)		1 (2.35)	
**Apgar Score at 1st Minute, *n* (%)**				0.744		0.846
0–3	42 (23.86)	16 (20.78)	26 (26.26)		11 (25.58)	
4–7	58 (32.95)	28 (36.37)	30 (30.30)		11 (25.58)	
8–10	76 (43.18)	33 (42.86)	43 (43.43)		21 (48.84)	
**Apgar Score at 5th Minute, *n* (%)**				0.179		0.382
0–3	17 (12.98)	5 (7.81)	12 (17.91)		5 (17.24)	
4–7	48 (36.64)	24 (37.50)	24 (35.82)		10 (34.48)	
8–10	66 (50.38)	35 (54.69)	31 (46.27)		14 (48.28)	
**Neonatal Convulsions, *n* (%)**				**0.001**		**0.001**
Yes	46 (26.90)	11 (14.47)	35 (36.84)		17 (40.48)	
No	125 (73.10)	65 (85.53)	60 (63.16)		25 (59.52)	
**Respiratory Failure, *n* (%)**				0.163		0.113
Yes	112 (69.13)	45 (63.38)	67 (73.63)		29 (78.38)	
No	50 (30.86)	26 (36.62)	24 (26.37)		8 (21.62)	
**Intraventricular Bleeding, *n* (%)**				0.907		0.811
Yes	95 (58.64)	42 (59.15)	53 (58.24)		21 (56.76)	
No	67 (41.36)	29 (40.85)	38 (41.76)		16 (43.24)	
**Infections in neonatal period, *n* (%)**				0.207		0.174
Yes	103 (63.19)	41 (57.75)	62 (67.39)		27 (71.05)	
No	60 (36.81)	30 (42.25)	30 (32.61)		11 (28.95)	

CP—cerebral palsy; ELBW—extremely low birth weight; VLBW—very low birth weight; LBW—low birth weight; * in comparison to CP patients without epilepsy.

## References

[1] Bax M., Goldstein M., Rosenbaum P., Leviton A., Paneth N., Dan B., Jacobsson B., Damiano D. (2005). Executive Committee for the Definition of Cerebral Palsy. Proposed definition and classification of cerebral palsy, April 2005. Dev. Med. Child Neurol..

[2] Rosenbaum P., Paneth N., Leviton A., Goldstein M., Bax M., Damiano D., Dan B., Jacobsson B. (2007). A report: The definition and classification of cerebral palsy April 2006. Dev. Med. Child Neurol..

[3] Cans C.H., Dolk H., Platt M.J., Colver A., Prasauskiene A., Krageloh-Mann I., SCPE Collaborative Group (2007). Recommendations from the SCPE collaborative group for defining and classifying cerebral palsy. Dev. Med. Child Neurol..

[4] Surveillance of Cerebral Palsy in Europe (2000). Surveillance of cerebral palsy in Europe: A collaboration of cerebral palsy surveys and registers. Dev. Med. Child Neurol..

[5] Platt M.J., Cans C., Johnson A., Surman G., Topp M., Torrioli M.G., Krageloh-Mann I. (2007). Trends in cerebral palsy among infants of very low birthweight (<1500 g) or born prematurely (<32 weeks) in 16 European centres: A database study. Lancet.

[6] Oskoui M., Coutinho F., Dykeman J., Jette N., Pringsheim T. (2013). An update on the prevalence of cerebral palsy: A systematic review and meta-analysis. Dev. Med. Child Neurol..

[7] Reddihough D.S., Collins K.J. (2003). The epidemiology and causes of cerebral palsy. Aust. J. Physiother..

[8] McIntyre S., Taitz D., Koegh J., Goldsmith S., Badawi N., Blair E. (2013). A systematic review of risk factors for cerebral palsy in children born at term in developed countries. Dev. Med. Child Neurol..

[9] Trabacca A., Vespino T., Di Liddo A., Russo L. (2016). Multidisciplinary rehabilitation for patients with cerebral palsy: Improving long-term care. J. Multidiscip. Health.

[10] Kwong K.L., Wong S.K., So K.T. (1998). Epilepsy in children with cerebral palsy. Pediatr. Neurol..

[11] Gururaj A.K., Sztriha L., Bener A., Dawodu A., Eapen V. (2003). Epilepsy in children with cerebral palsy. Seizure.

[12] Kułak W., Sobaniec W. (2003). Risk factors and prognosis of epilepsy in children with cerebral palsy in north-eastern Poland. Brain Dev..

[13] Mert G.G., Incecik F., Altunbasak S., Herguner O., Mert M.K., Kiris N., Unal I. (2011). Factors affecting epilepsy development and epilepsy prognosis in cerebral palsy. Pediatr. Neurol..

[14] Wallace S.J. (2001). Epilepsy in cerebral palsy. Dev. Med. Child Neurol..

[15] Marszał E. (2006). Incidence, diagnostics and treatment of epilepsy in children with cerebral palsy. Neurol. Dziec..

[16] Jekovec-Vrhovsek M. (2012). Epilepsy in children with cerebral palsy. East. J. Med..

[17] Zafeiriou D.I., Kontopoulos E.E., Tsikoulas I. (1999). Characteristics and prognosis of epilepsy in children with cerebral palsy. J. Child Neurol..

[18] Bruck I., Antoniuk S.A., Spessatto A., Bem R.S., Hausberger R., Pacheco C.G. (2001). Epilepsy in children with cerebral palsy. Arq. Neuropsiquiatr..

[19] Sellier E., Uldall P., Calado E., Sigurdardottir S., Torrioli M.G., Platt M.J., Cans C.H. (2012). Epilepsy and cerebral palsy: Characteristics and trends in children born in 1976-1998. Eur. J. Paediatr. Neurol..

[20] Balf C.L., Ingram T.T. (1955). Problems in the Classification of Cerebral Palsy in Childchood. Br. Med. J..

[21] Palisano R.J., Cameron D., Resenbaum P.L., Walter S.D., Rusell D. (2006). Stability of the gross motor function classification system. Dev. Med. Child Neurol..

[22] Himmelmann K., Horber V., De la Cruz J., Horridge K., Mejaski-Bosnjak V., Hollody K., Krageloh-Mann I. (2017). on befalf of the SCPE Working Group. MRI classification system (MRICS) for children with cerebral palsy: Development, reliability, and recommendations. Dev. Med. Child Neurol..

[23] Fisher R.S., van Emde Boas W., Blume W., Elger C., Genton P., Lee P., Elgen J. (2005). Epileptic Seizures and Epilepsy: Definitions Proposed by the International League against Epilepsy (ILAE) and the International Bureau for Epilepsy (IBE). Epilepsia.

[24] Fisher R.S., Acevedo C., Arzimanoglou A., Bogacz A., Cross J.H., Elger C.H.E., Engel J., Forsgren L., French J.A., Glynn M. (2014). ILAE official report: A practical clinical definition of epilepsy. Epilepsia.

[25] Kwan P., Arzimanoglou A., Berg A.T., Brodie M.J., Hauser W.A., Mathern G., Moshe S.L., Perucca E., Wiebe S., French J. (2010). Definition of drug resistant epilepsy: Consensus proposal by the ad hoc Task Force of the ILAE Commission on Therapeutic Strategies. Special Report. Epilepsia.

[26] Tellez-Zenteno J.F., Hernandez-Ronquillo L., Buckley S., Zahagun R., Rizvi S. (2014). A validation of the new definition of drug-resistant epilepsy by the International League against Epilepsy. Epilepsia.

[27] Lopez Gonzalez F.J., Rodriguez Osorio X., Gil- Nagel Rein A., Carreno Martinez M., Serratosa Fernandez J., Villanueva Haba V., Donaire Pedraza A.J., Mercade Cerda J.M. (2015). Drug-resistant epilepsy: Definition and treatment alternatives. Neurologia.

[28] Surveillance of Cerebral Palsy in Europe (2002). Prevalence and characteristics of children with cerebral palsy in Europe. Dev. Med. Child Neurol..

[29] Mieszczanek T. (2003). Selected epidemiologic aspects of cerebral palsy in the population of children and adolescents in west-east Poland. Neurol. Dziec..

[30] Kułak W., Sobaniec W., Okurowska-Zawada B., Sienkiewicz D., Paszko-Patej G. (2009). Antenatal, intrapartum and neonatal risk factors for cerebral palsy in children in Podlaskie Province. Neurol. Dziec..

[31] Kułak W., Sobaniec W., Śmigielska-Kuzia J., Boćkowski L. (2006). Neurophysiologic and neuroimaging studies of brain plasticity in children with cerebral palsy. Exp. Neurol..

[32] Hadjipanayis A., Hadjichristodoulou C., Youroukos S. (1997). Epilepsy in patients with cerebral palsy. Dev. Med. Child Neurol..

[33] Delacy M.J., Reid S.M. (2016). Profile of associated impairments at age 5 years in Australia by cerebral palsy subtype and Gross Motor Function Classification System level for birth years 1996 to 2005. Dev. Med. Child Neurol..

[34] Turkoglu G., Turkoglu S., Celik C., Ucan H. (2017). Intelligence, Functioning and Related Factors in Children with Cerebral Palsy. Arch. Neuropsychiatry.

[35] Sigurdardottir S., Eiriksdottir A., Gunnarsdottir E., Meintema M., Arnadottir U., Vik T. (2008). Cognitive profile in young Icelandic children with cerebral palsy. Dev. Med. Child Neurol..

[36] Reid S.M., Meehan E.M., Arnup S.J., Reddihough D.S. (2018). Intellectual disability in cerebral palsy: A population- based retrospective study. Dev. Med. Child Neurol..

[37] Carlsson M., Hagberg G., Olsson I. (2003). Clinical and aetiological aspects of epilepsy in cerebral palsy. Dev. Med. Child Neurol..

[38] Delgado M.R., Riela A.R., Mills J., Pitt A., Browne R. (1996). Discontinuation of antiepileptic drug treatment after two seizure- free years in children with cerebral palsy. Pediatrics.

[39] Zelnik N., Konopnicki M., Bennett-Back O., Castel-Deutsch T., Tirosh E. (2010). Risk factors for epilepsy in children with cerebral palsy. Eur. J. Paediatr. Neurol..

[40] Douglass L.M., Heeren T.C., Stafstrom C.E., DeBassio W., Allred E.N., Leviton A., O’Shea T.M., Hirtz D., Rollins J., Kuban K. (2017). Cumulative Incidence of Seizures and Epilepsy in Ten-Year- Old Children Born Before 28 Weeks’ Gestation. Pediatr. Neurol..

[41] Persson M., Razaz N., Tedroff K., Joseph K.S., Cnattingius S. (2018). Five and 10 minute Apgar scores and risks of cerebral palsy and epilepsy: Population based cohort study in Sweden. BMJ.

[42] Wu C.S., Sun Y., Vestergaard M., Christensen J., Ness R.B., Haggerty C.L., Olsen J. (2008). Preeclampsia and risk for epilepsy in offspring. Pediatrics.

[43] Razaz N., Tedroff K., Villamor E., Cnattingius S. (2017). Maternal Body Mass Index in Early Pregnancy and Risk of Epilepsy in Offspring. JAMA Neurol..

[44] O’Callaghan M., MacLennan A. (2013). Cesarean Delivery and Cerebral Palsy: A Systematic Review and Meta-analysis. Obstet. Gynecol..

[45] Ehrenstein V., Pedersen L., Holsteen V., Larsen H., Rothman K.J., Sørensen H.T. (2007). Postterm delivery and risk for epilepsy in childhood. Pediatrics.

[46] Deoni S.C., Adams S.H., Li X., Badger T.M., Pivik R.T., Glasier C.M., Ramakrishnaiah R.H., Rowell A.C., Ou X. (2019). Cesarean Delivery Impacts Infant Brain Development. AJNR Am. J. Neuroradiol..

